# Metabolomic and transcriptomic analyses provide insights into the red pigmentation in loquat (*Eriobotrya japonica*) peel

**DOI:** 10.3389/fpls.2025.1615281

**Published:** 2025-06-18

**Authors:** Yaling Zhang, Xiuping Chen, Wenbing Su, Chaojun Deng, Jimou Jiang, Shaoquan Zheng

**Affiliations:** Fruit Research Institute, Fujian Academy of Agricultural Sciences, Fujian, Fuzhou, China

**Keywords:** loquat, RNA-seq, anthocyanin biosynthesis, EjMYB10, anthocyanin-targeted metabolome

## Abstract

Loquat (*Eriobotrya japonica* Lindl.) is a subtropical evergreen tree native to China. Generally, the pigments accumulated in the fruits of cultivated loquats are carotenoids rather than anthocyanins. In this study, we showed that the peel of ‘Shanpaisanhao’ loquats can accumulate anthocyanins and turn red. We further investigated the mechanisms underlying anthocyanin accumulation in the red-pigmented peels of ‘Shanpaisanhao’ loquats. RNA-seq analysis demonstrated that anthocyanin accumulation in loquat peel is associated with the upregulation of anthocyanin biosynthetic and transport genes as well as the transcriptional factor *EjMYB10.* Transient overexpression and dual luciferase assays showed that *EjMYB10* could induce weak anthocyanin accumulation in tobacco leaves when co-expressed with *PsbHLH3*, and activate the promoters of *EjF3′H*, *EjANS* and *EjUFGT*. These results provide information for further elucidating the molecular mechanism of anthocyanin accumulation in the peel of SP3H loquat and for breeding of new red-pigmented loquat cultivars.

## 1 Introduction

Loquat (*Eriobotrya japonica* Lindl.) is a subtropical fruit tree native to China and is cultivated commercially worldwide nowadays. Generally, fruits of cultivated loquats only accumulate carotenoids, the pigments that endow mature fruits with yellow-, orange-, or orange-red color (Zhou et al., 2007), and do not accumulate anthocyanins. Interestingly, the fruit peels of some *Eriobotrya* species, such as *E. henryi* and *E. seguinii*, are red or purple, which does not seem to be a result of carotenoid accumulation. Our previous study showed that the peel of *E. henryi* fruits appears red color due to the accumulation of anthocyanins (Su et al., 2023). It will be interesting to examine whether other *Eriobotrya* species accumulate anthocyanins in their fruits.

Anthocyanins are natural pigments that are responsible for the red coloration of fruits. Anthocyanins are produced by a branch of the flavonoid biosynthetic pathway that has been well documented in plants. The anthocyanin biosynthetic enzymes include phenylalanine ammonia-lyase (PAL), cinnamate-4-hydroxylase (C4H), 4-coumaroyl:CoA-ligase (4CL), chalcone synthase (CHS), chalcone isomerase (CHI), flavanone 3-hydroxylase (F3H), flavonoid 3′-hydroxylase(F3′H), flavonoid 3′,5′-hydroxylase, dihydroflavonol 4-reductase (DFR), anthocyanidin synthase/leucoanthocyanidin dioxygenase (ANS/LDOX) and UDP-glucose:flavonoid3-O-glucosyltransferase (UFGT) (Jaakola, 2013; Tanaka et al., 2008). Finally, anthocyanins are transported to and deposited in the vacuole with the assistance of transporters such as glutathione S-transferase (GST) (Zhao and Dixon, 2010). Su et al. (2023) demonstrated that *PAL*, *4CL*, *CHS*, *CHI*, *F3H*, *F3′H*, *DFR*, *ANS* and *UFGT* were highly expressed in the peel of *E. henryi* fruits that accumulate anthocyanins. These results implicate that anthocyanin accumulation in loquat fruits was transcriptionally regulated.

Numerous evidence demonstrates that MYB-bHLH-WD40 (MBW) complex is the key component of the transcriptional regulatory network of anthocyanin accumulation (Allan et al., 2008; Zhao et al., 2023) and many transcriptional factors were shown to modulate anthocyanin accumulation through interactions with the MBW complex (Yan et al., 2021; Yang et al., 2022). In rosaceous species, R2R3 MYB activators have been identified as key anthocyanin regulators (Espley et al., 2007; Fang et al., 2021b; Feng et al., 2015; Gu et al., 2015; Lin-Wang et al., 2010; Ravaglia et al., 2013). However, transcription factors that regulate anthocyanin accumulation in loquat fruits remain to be identified. Lin-Wang et al. (2010) isolated the genomic DNA of *MYB10* from rosaceous crop species, including loquat (*Eriobotrya japonica*) and showed that these sequences were highly conserved. Whether *EjMYB10* is expressed and functions in anthocyanin biosynthesis remains to be tested.

We found that ‘Shanpaisanhao’ (SP3H, *E. japonica* Lindl.) loquat fruits turn red under certain conditions. However, it is unclear whether anthocyanins are the main pigments in the red pigmented peels and the underlying mechanism remains to be elucidated. In this study, we showed that anthocyanins were the red pigments accumulated in the peel of SP3H loquat fruits. RNA-seq results showed that the transcription of anthocyanin pathway genes and transcription factor *EjMYB10* were upregulated in red pigmented peels. Transient expression of *EjMYB10* induced anthocyanin production in tobacco leaves. Furthermore, dual luciferase assays showed that *EjMYB10* activated the promoters of *EjF3′H*, *EjANS* and *EjUFGT*. This study suggests that *EjMYB10* acts as an anthocyanin activator and is responsible for anthocyanin accumulation in the peel of SP3H loquat.

## 2 Materials and methods

### 2.1 Plant materials

Fruits of the SP3H loquat with obvious red pigmentation were collected from the National Loquat Germplasm Bank (Fuzhou, Fujian, China) and used for further analysis. Unpigmented and red-pigmented peels were collected and sliced into small pieces. Three biological replicates were prepared, with ten fruits were included in each replicate. The peels of each replicate were pooled and rapidly frozen in liquid nitrogen and stored at -80°C until use.

### 2.2 Anthocyanin extraction and targeted metabolome analysis

Peel samples were subjected to anthocyanin-targeted metabolome analysis to identify anthocyanins accumulated in red-pigmented peels. All procedures of the metabolome analysis were performed by Metware Biotechnology Co., Ltd. (Wuhan, China). Loquat peels were freeze-dried using a vacuum freeze-dryer and then ground into fine powder using a mixer mill. Anthocyanins were extracted and subjected to UPLC-MS/MS analysis as described by Fang et al. (2025). Briefly, 50 mg of powder was dissolved in 0.5 mL of extraction solution (methanol: water: hydrochloric acid=500: 500: 1, V/V/V) and the mixture was vortexed for 5 min. Then the mixture was sonicated for 5 min and centrifuged at 12, 000 g at 4°C for 3 min. The supernatant was collected and the residue was re-extracted as described above. The supernatants were pooled and filtered through a 0.22 μm pore size filter prior to LC-MS/MS analysis. UPLC-MS/MS analysis was conducted using an UPLC-ESI-MS/MS system (UPLC, ExionLC™ AD; MS, Applied Biosystems 6500 Triple Quadrupole). The parameters were as follows: the column employed was a Waters ACQUITY BEH C18 (1.7 μm, 2.1 mm×100 mm); The mobile phase was a mixture of water (0.1% formic acid): methanol(0.1% formic acid); The gradient elution was 95:5 V/V for 6 min, 50:50 V/V for 6 min, 5:95 V/V for 2 min, and then returned to 95:5 V/V for 2 min; The flow rate was 0.35 mL/min, and the temperature was maintained at 40°C; The volume of injected sample was 2 μL.

### 2.3 Transcriptome sequencing and analysis

Extraction of total RNA from loquat peels and library construction were carried out at Beijing BioMarker Technologies (Beijing, China) as described in our previous study (Su et al., 2023). cDNA libraries were sequenced using Illumina NovaSeq 6000. RNA-seq was performed in three biological replicates. The RNA-seq reads have been deposited in the Genome Sequence Archive (GSA) (Chen et al., 2021) of the National Genomics Data Center (Members and Partners, 2024) (https://ngdc.cncb.ac.cn/gsa) and are accessible under PRJCA038065. The obtained raw 150 bp paired-end reads were processed as described previously (Su et al., 2023) and mapped to the genome of ‘Jiefangzhong’ (Su et al., 2021) using HISAT2. DESeq2 was used to calculate gene expression level and identify differentially expressed genes (DEGs). Only genes that met with the criteria of fold change >2 and a false discovery rate (FDR) <0.01 were selected as DEGs. GO and KEGG pathway enrichment analyses was performed as described previously (Su et al., 2023). Prediction of transcription factors was performed using the Transcription Factor Prediction module of PlantTFDB v5.0 (https://planttfdb.gao-lab.org/prediction.php).

### 2.4 qRT-PCR (quantitative real-time PCR) analysis

Total RNA was extracted from loquat peels using the EZNA Plant RNA Kit (Omega Bio-tek). qRT-PCR was performed as described previously (Zhang et al., 2021). *EjActin* (Ej00095133) was used as the reference gene. A LightCycler 480 real-time PCR system was employed for qRT-PCR analysis, with three biological and four technical replicates. The primer sequences used in qRT-PCR are listed in **Supplementary Table S1**.

### 2.5 Vector construction

cDNA from red pigmented SP3H loquat peels was synthesized using the HiScript III 1st Strand cDNA Synthesis Kit (Vazyme, Nanjing, China). The isolation of *EjMYB10* coding sequence and insertion into pSAK277 were carried out using the 2×Phanta Max Master Mix (Vazyme, Nanjing, China) and the ClonExpress Ultra One Step Cloning Kit (Vazyme, Nanjing, China), respectively. The promoter of *EjF3′H*, *EjANS* and *EjUFGT* were amplified and cloned into pGreenII 0800-LUC to generate reporter construct as described previously (Zhang et al., 2021) and their sequences are provided in **Supplementary Table S2**. All primers used for cloning are listed in **Supplementary Table S3**.

### 2.6 Phylogenetic analysis and sequence alignment

Phylogenetic analyses were performed using the neighbor-joining method with 1000 bootstrap replicates by MEGA6. The alignment of amino acid sequences was carried out using ClustalW (https://www.genome.jp/toolsbin/clustalw). ESPript 3.0 (Robert and Gouet, 2014) was employed to shade the results of the multiple sequence alignment. The accession numbers of additional sequences from other species are provided in **Supplementary Table S4**.

### 2.7 Transient expression in tobacco leaf and determination of total anthocyanin content

Transient expression of *EjMYB10* was carried out in *Nicotiana tabacum* young leaves as described previously (Zhang et al., 2021). Basic helix-loop-helix (bHLH) transcription factors that belong to the bHLH2 subgroup (PhAN1/AtTT8) have been suggested as indispensable partners of anthocyanin-promoting R2R3-MYBs (Espley et al., 2007; Gonzalez et al., 2008). These bHLHs are required for anthocyanin biosynthesis in tobacco leaves (Montefiori et al., 2015). Sequence analysis showed that *Ej00089823* (*EjbHLH3*) was predicted to encode a bHLH2 subgroup bHLH protein. EjbHLH3 was highly homologous to apple MdbHLH3 (Xie et al., 2012), peach PpbHLH3 (Ravaglia et al., 2013) and plum PsbHLH3 (Fang et al., 2021a) (**Supplementary Figure S1**). We failed to clone *EjbHLH3*, so we chose the plum *PsbHLH3* which was sufficient to induce anthocyanin accumulation in tobacco leaves when coinfiltrated with plum MYB10s (Fang et al., 2021a, Fang et al., 2021b) and blueberry VcMYBA (Zhang et al., 2021). Agrobacteria carrying constructs were cultivated and resuspended in the infiltration buffer (10 mM MES, 10 mM MgCl_2_, and 100 μM acetosyringone) to an OD_600_ of 0.6 and then infiltrated into tobacco leaves. Photos of transformed tobacco leaves were taken 7 d after infiltration. Anthocyanin in tobacco leaves was extracted and quantified as described by Fang et al. (2025).

### 2.8 Dual-luciferase assay

pGreenII 0800-LUC vectors carrying the promoter of *EjF3′H*, *EjANS* and *EjUFGT* were transformed into the *A. tumefacien* strain GV3101 with pSoup. Dual-luciferase assays were performed using 3 to 4-week-old *Nicotiana benthamiana* leaves. Agrobacteria were grown and resuspended in the infiltration buffer to an OD_600_ of 0.5 and then used for infiltration according to the protocol described above for transient expression assays. Firefly luciferase (LUC) activity detection and image capture were performed 3d after infiltration, as described by Fang et al. (2025). The detection of firefly luciferase and *Renilla* luciferase activities was carried out using a GloMax Discover instrument (Promega) and the Dual Luciferase Reporter Gene Assay Kit (Yeasen, 11402ES80, China).

### 2.9 Statistical analysis

Statistical analysis was performed using Student’s t-test (*P < 0.05, **P < 0.01, ***P<0.001 and ****P<0.0001). Data analysis was performed using GraphPad Prism 10.3.1 and TBtools-II (Chen et al., 2023).

## 3 Results

### 3.1 Anthocyanin accumulation in the peel of SP3H loquat fruits

**Figure 1:**
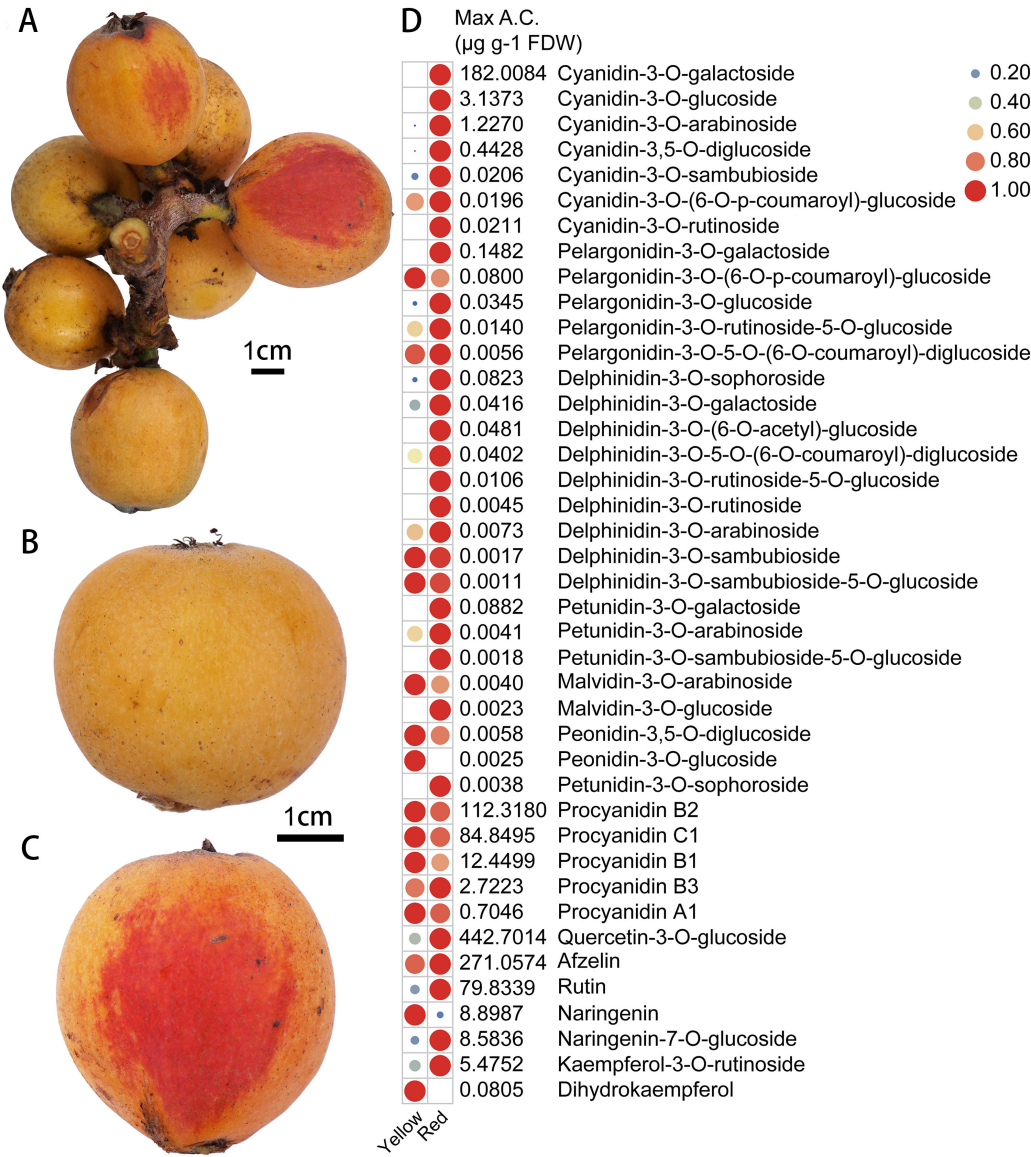
Anthocyanin accumulation in the peel of SP3H loquat. **(A)** fruits of SP3H loquat. **(B)** normal SP3H loquat fruit. **(C)** red pigmented SP3H loquat fruit. **(D)** heatmap of flavonoids identified in the peel of SP3H loquat. Each value is the average for three biological replicates. The contents of flavonoids were normalized with the maximum content values of each flavonoid. The content of flavonoids is indicated using filled circles of different sizes and colors. The larger the circle, the higher the expression. The values on the right indicate the highest content value of each flavonoid.

Generally, the ripe SP3H loquat fruits are yellow. However, partial red pigmentation was found in the peel of some ‘Shanpaisanhao’ loquat fruits (**Figures 1A–C**). Our previous study showed that anthocyanins are the main pigments accumulated in red loquat peels (Su et al., 2023). Flavonoids metabolomics analysis was performed to investigate the composition of red pigments in the red pigmented areas. In total, 41 flavonoid metabolites, including seven cyanidin, five pelargonidin, nine delphinidin, five petunidin, two peonidin and two malvidin derivatives were identified (**Figure 1D**). 21 of the identified anthocyanins were significantly accumulated in the red-pigmented areas, and cyanidin-3-O-galactoside was the most predominant anthocyanin (**Figure 1D**). In addition, the accumulation of rutin, naringenin-7-O-glucoside and quercetin-3-O-glucoside were also detected in the red pigmented areas (**Figure 1C**). These results suggest that anthocyanin accumulation is responsible for the red pigmentation in the peel of SP3H loquat fruits.

### 3.2 RNA-seq and identification of differentially expressed genes

The yellow and red pigmented peels of SP3H loquat fruits were further subjected to RNA-seq analysis to explore the mechanism of anthocyanin biosynthesis. A total of 38.17 Gb clean data was obtained from loquat peel samples (**Supplementary Table S5**). Over 94% of the obtained reads could be mapped to the genome of ‘Jiefangzhong’ (**Supplementary Table S5**). In total, 10072 transcripts were detected in analyzed peel samples. These include 2429 putative new genes that have not been predicted in the genome of ‘Jiefangzhong’ and 1624 of them was annotated by databases (**Supplementary Table S6**). Transcriptome comparison showed that 2821 genes were differentially expressed (1770 upregulated and 1051 downregulated) between yellow and red pigmented areas.

### 3.3 Identification of differentially expressed genes involved in anthocyanin accumulation

**Figure 2:**
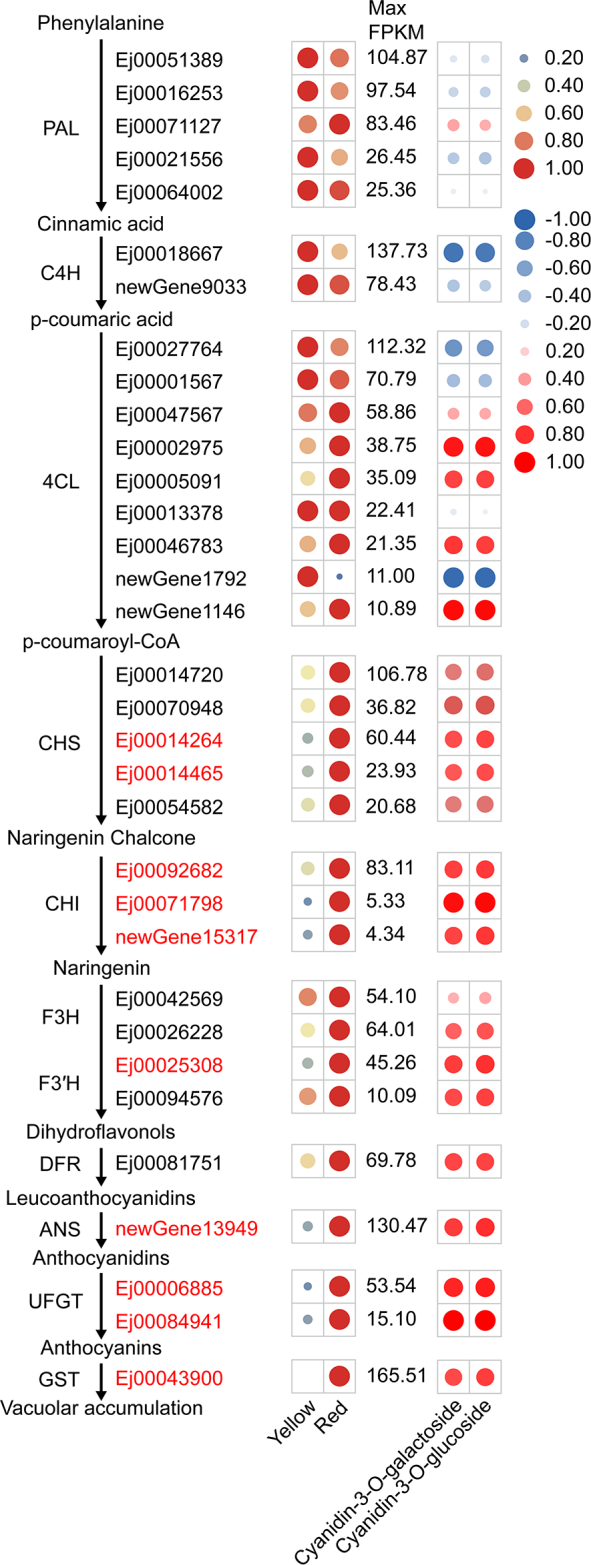
Expression of structural genes involved in anthocyanin biosynthesis, as evaluated by RNA-sequencing in the peel of SP3H loquat. The left panel indicated anthocyanin pathway including gene IDs, and the expression profile of structural genes. FPKM values of all genes were normalized with maximum FPKM values of each gene. The expression level of genes was indicated using filled circle with different size and color. The larger the circle, the higher the expression. High expression was indicated in red, while low expression was indicated in green. The values on the middle indicates the highest FPKM value of each gene. Abbreviations for pathway genes as described in the Introduction. The right panel indicated the correlation between structural genes and anthocyanin content. The correlation coefficient was indicated using filled circle with different size and color. Positive correlation was indicated in red, while negative correlation was indicated in blue.

KEGG enrichment analysis of DEGs indicated that 52 DEGs were involved in phenylpropanoid biosynthesis, flavonoid biosynthesis and anthocyanin biosynthesis pathways (**Supplementary Table S7**). Nine of these genes, including *CHS* (Ej00014264 and Ej00014465), *CHI* (Ej00092682, Ej00071798 and newGene15317), *F3′H* (Ej00025308), *ANS* (newGene13949) and *UFGT* (Ej00006885 and Ej00084941) were assigned to the anthocyanin biosynthetic pathway (**Figure 2**). The expression of these genes was positively correlated (r>0.65) with the cyanidin-3-O-galactoside and cyanidin-3-O-glucoside concentration (**Figure 2**). Other anthocyanin pathway genes, including *4CL* (Ej00002975, Ej00005091, Ej00046783 and newGene1146), *CHS* (Ej00014720, Ej00070948 and Ej00054582), *F3H* (Ej00026228), *DFR* (Ej00081751), were not identified as differentially expressed genes, but their transcription was also enhanced in the red-pigmented peels. Additionally, the expression of a *glutathione S-transferase* (*GST*) gene Ej00043900 (annotated as GSTF12) is also positively correlated (r>0.7) with the concentrations of cyanidin-3-O-galactoside and cyanidin-3-O-glucoside (**Figure 2**). Phylogenetic analysis showed that Ej00043900 fell into a same clade with anthocyanin-related GSTs and was closely related to anthocyanin-related GST Raint from peach (**Supplementary Figure S2**). These results suggested that anthocyanin biosynthesis in peel of SP3H loquats is regulated by the transcription of these structural genes.

**Figure 3:**
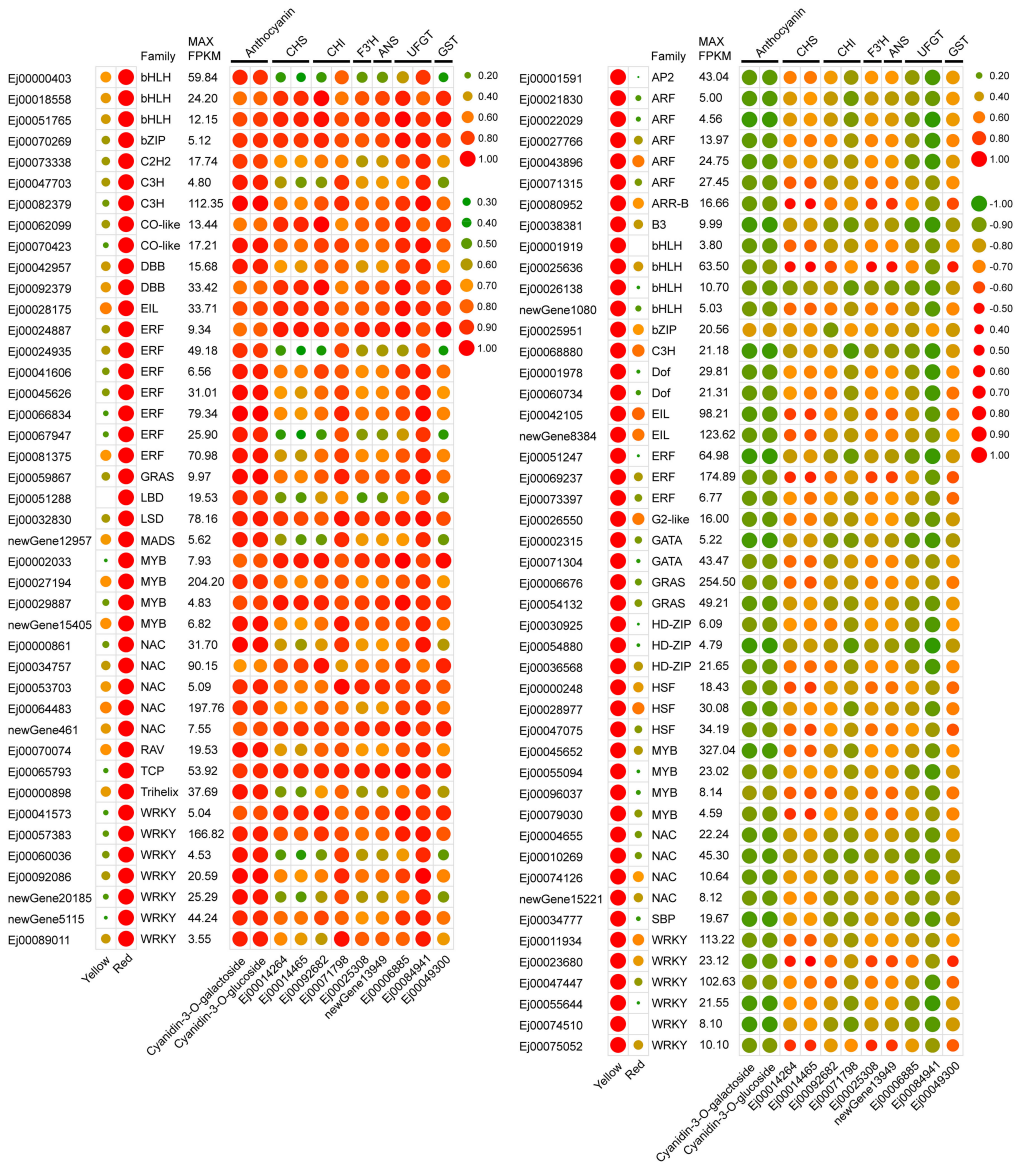
Expression profiles of selected differentially expressed transcription factors. Left panel showed expression profiles of expressed transcription factors. Right panel showed the correlation coefficient between the expression of transcription factors and structural genes or anthocyanin content.

The obvious upregulation of anthocyanin biosynthetic genes *CHS*, *CHI*, *F3′H* and *ANS* and anthocyanin transportation gene *GSTF12* in red-pigmented peels suggests that anthocyanin accumulation in the SP3H loquat peel was transcriptionally regulated. To identify candidate transcriptional regulators that regulate anthocyanin accumulation, the sequences of loquat genes were submitted to PlantTFDB v5.0 to predict transcription factors. In total, 2365 genes were identified to encode transcriptional factor. We further analyzed the expression patterns of the identified transcription factors, and the results showed that 208 of them were differentially expressed (with maximum FPKM values ≥ 5, **Supplementary Table S8**). Among these differentially expressed transcription factors, 42 were positively correlated (r > 0.9) and 47 were negatively correlated (r|< -0.9) were positively and negatively correlated with the concentrations of cyanidin-3-O-galactoside and cyanidin-3-O-glucoside or structural genes, respectively (**Figure 3**).

### 3.4 Expression analysis of candidate anthocyanin-related genes by qRT-PCR

**Figure 4:**
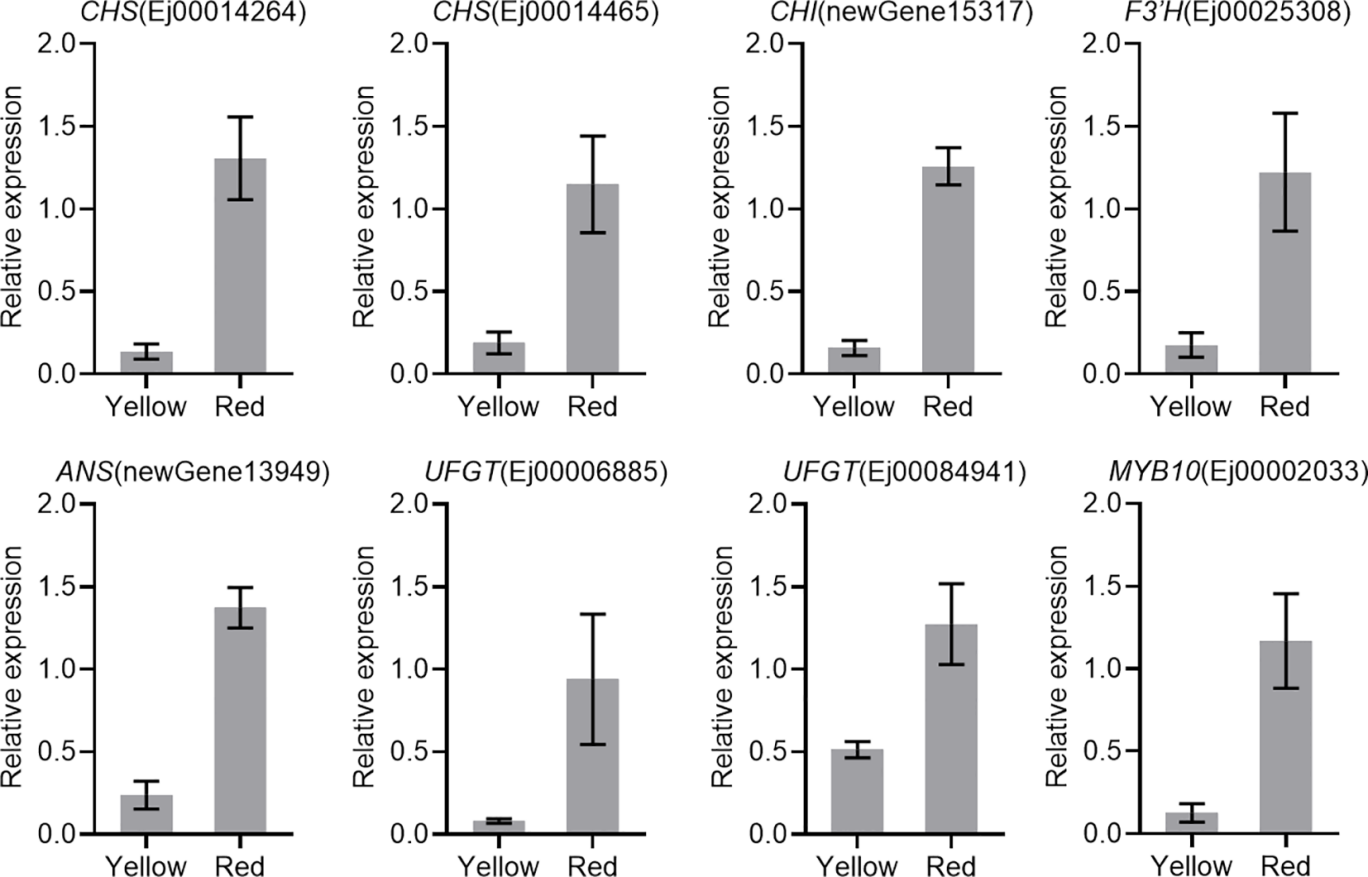
Expression of candidate genes involved in anthocyanin biosynthesis. *Actin* was used as the internal control. The error bars represent the standard errors of three biological replicates.

The results of RNA-Seq were validated by analyzing the expression profiles of eight candidate anthocyanin-related genes, including six anthocyanin biosynthetic genes (*CHS*, *CHI*, *F3′H*, *ANS* and *UFGT*), one *GST* gene, and one *MYB*, using qRT-PCR. The results showed that all analyzed genes were upregulated in the red-pigmented peel (**Figure 4**).

### 3.5 *EjMYB10* is a positive regulator of anthocyanin biosynthesis

**Figure 5:**
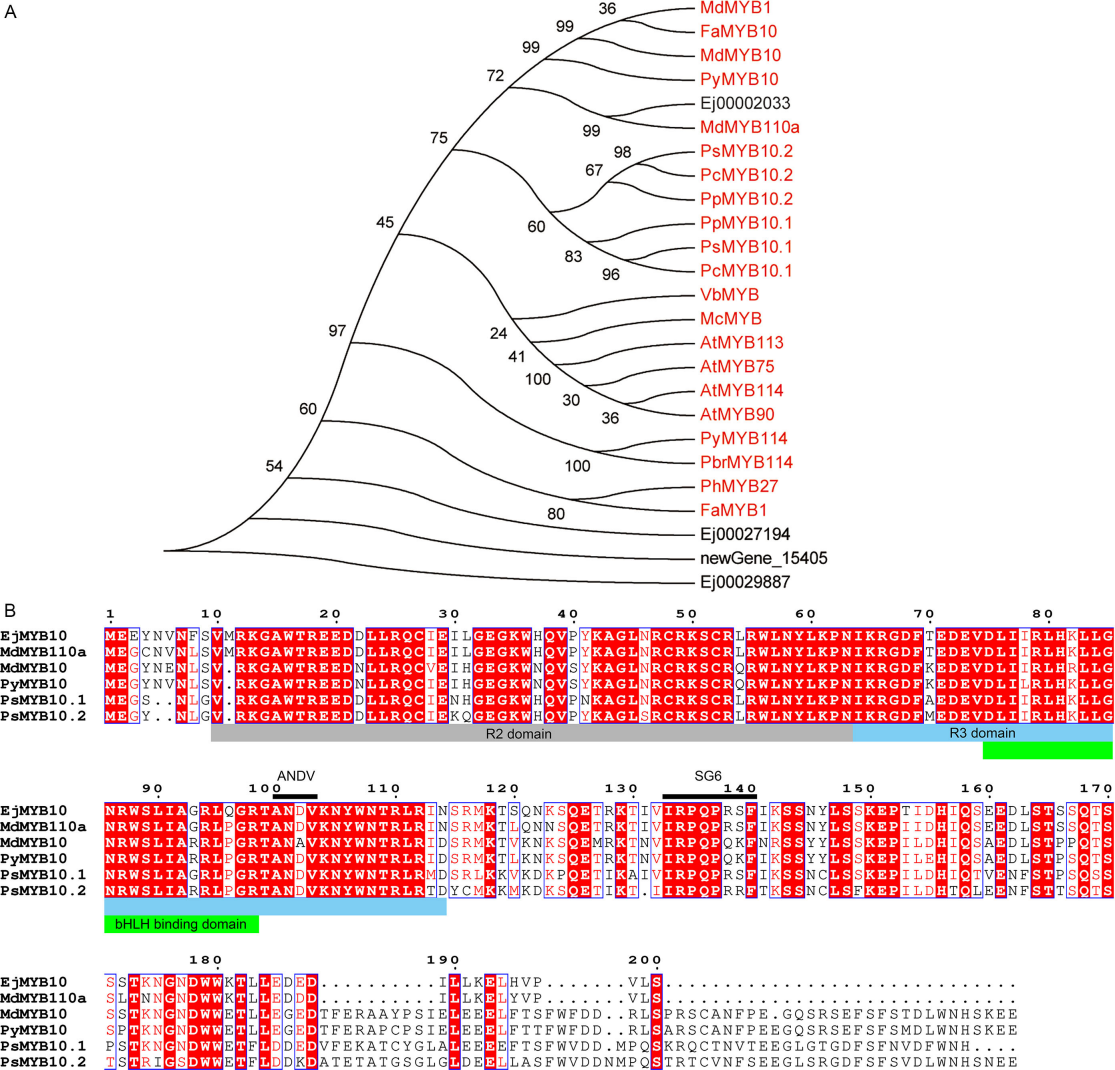
Sequence analysis of selected EjMYBs. **(A)** phylogenetic analysis of EjMYBs and R2R3 MYB activators from other plant species. **(B)** sequence alignment of EjMYB10 and anthocyanin MYB activators from other plant species. R2, R3, and bHLH binding domain are highlighted in gray, blue and green boxes, conserved ‘ANDV’ and SG6 motif for anthocyanin-promoting MYBs are indicated under black lines.

MYBs serve as crucial regulators of anthocyanin biosynthesis. Our results demonstrated that four MYBs (Ej00002033, Ej00027194, Ej00029887 and newGene_15405) exhibited an expression profile that was positively correlated with anthocyanin concentration and the expression pattern of structural genes of anthocyanin biosynthesis pathway (**Figure 3**). Phylogenetic analysis showed that Ej00002033 (*EjMYB10*) encodes a MYB that fell into the same clade with MYB10s from other rosaceous species and was closely related to apple MdMYB110a (**Figure 5A**; **Supplementary Table S**). Multiple sequence alignment revealed EjMYB10 was highly homologous to MdMYB110a and a conserved R2R3 domain as well as the ‘ANDV’ and SG6 motifs, which are characteristic of anthocyanin-promoting MYBs (**Figure 5B**). However, the C-terminal of EjMYB10 protein is shorter than that of MdMYB10, PyMYB10, PsMYB10.1, and PsMYB10.2 (**Figure 5B**).

**Figure 6:**
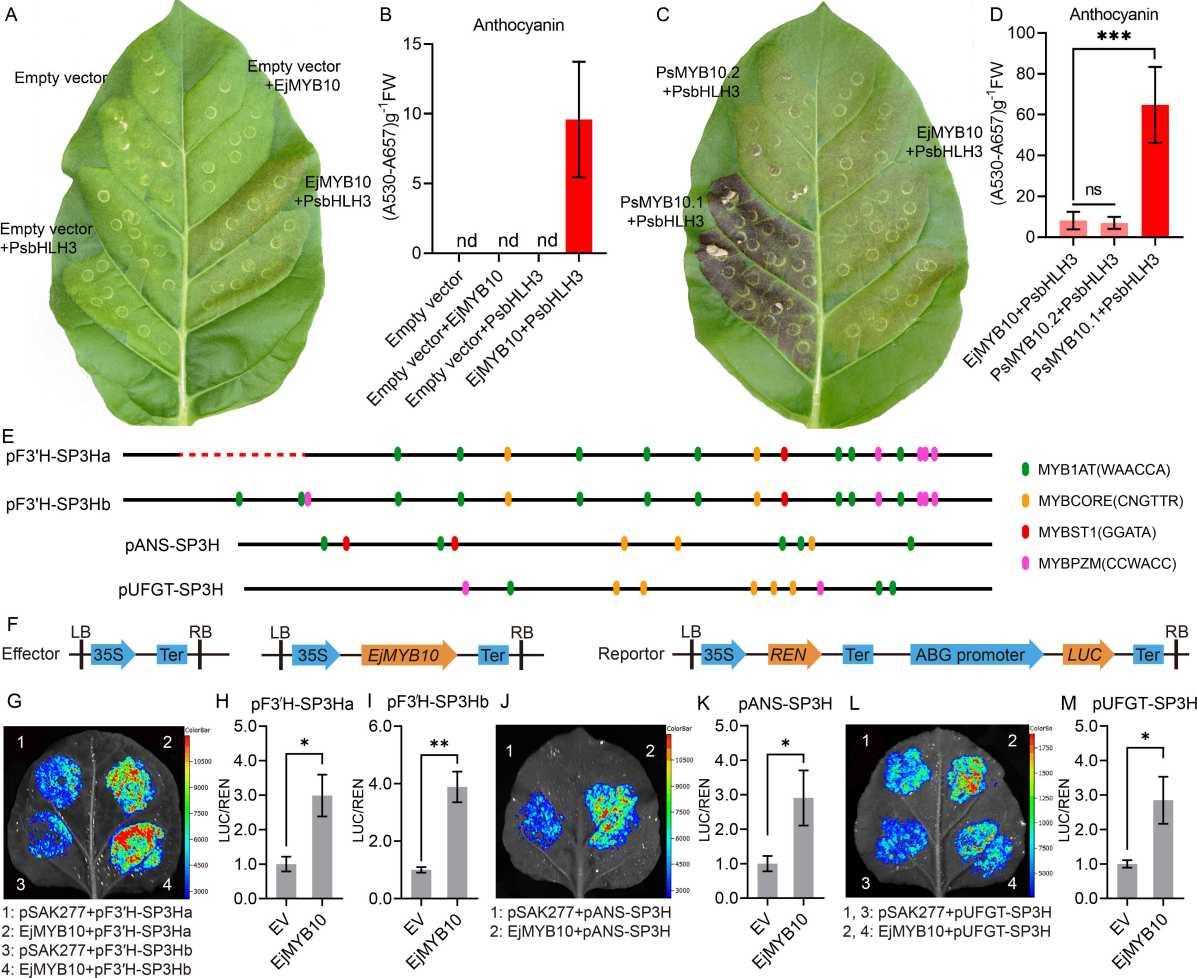
Functional analysis of the loquat *EjMYB10* gene. **(A, C)** transient color assay of the *EjMYB10* activity in tobacco leaf. **(B, D)** anthocyanin content in tobacco leaves. **(E)** schematic diagram of the *EjF3′H*, *EjANS* and *EjUFGT* promoter. The search of potential MYB-binding elements described by Zhou et al. (2019) was carried out using the PlantPAN4.0 (https://plantpan.itps.ncku.edu.tw/plantpan4/promoter_analysis.php). Color ellipses represent predicted MYB binding sites. Red dashed line indicated the location of sequence deletion in the promoter of *EjF3′H*. **(F)** schematic diagram showing the effector and reporter constructs for the dual-luciferase assay. **(G–M)** Validation of activation effect of *EjMYB10* on the *EjF3′H*, *EjANS* and *EjUFGT* promoters using dual luciferase assay. **(G, J, L)** Representative images of tobacco leaves 72 h after infiltration. **(H, I, K, M)** relative firefly LUC-to-REN ratios from transient expression assays. Asterisks denote t-test significance: *P < 0.05, **P < 0.01.

Transient expression in tobacco leaves was employed to validate the function of EjMYB10 in anthocyanin biosynthesis. Our results showed that faint red coloration was observed in leaves co-infiltrated with *EjMYB10* and *PsbHLH3* eight days after transformation. However, no red pigmentation was observed when *EjMYB10* or *PsbHLH3* was infiltrated alone (**Figure 6A**). Quantification of anthocyanins in tobacco leaves revealed that only a small amount of anthocyanin was accumulated in leaves infiltrated with *EjMYB10* and *PsbHLH3* (**Figure 6B**). In contrast, no anthocyanin was detected in leaves infiltrated with empty vector, *EjMYB10* or *PsbHLH3* (**Figure 6B**). These results suggest that EjMYB10 functions as a weak anthocyanin activator. To confirm this, the anthocyanin-promoting activity of *EjMYB10* was further compared with strong activator *PsMYB10.1* (Fang et al., 2021a) and weak activator *PsMYB10.2* (Fang et al., 2021b) from plum. Coinfiltration of *PsMYB10.1* with *PsbHLH3* led to obvious red pigmentation at infiltration sites three days after transformation, and strong anthocyanin accumulation was observed eight days after transformation (**Figure 6C**). However, the infiltration of *PsMYB10.2* and *PsbHLH3* or *EjMYB10* and *PsbHLH3* resulted in only weak red pigmentation in the leaves (**Figure 6C**). Our results showed that the anthocyanin content in tobacco leaves infiltrated with *EjMYB10* and *PsbHLH3* was comparable to that in leaves infiltrated with *PsMYB10.2* and *PsbHLH3*. However, it was significantly lower than the content in leaves infiltrated with *PsMYB10.1* and *PsbHLH3* (**Figure 6D**). Additionally, promoter structure analysis revealed that the promoters of the anthocyanin biosynthetic genes *EjF3′H* (Ej00025308), *EjANS* (newGene 13949) and *EjUFGT* (Ej00006885) contain multiple potential MYB binding sites (**Figure 6E**). Dual-luciferase assays were carried out to further verify whether EjMYB10 could interact with the promoters of these anthocyanin biosynthetic genes. Infiltration of *EjMYB10* was able to enhance the transcriptional activity of *EjF3′H*, *EjANS* and *EjUFGT* (**Figures 6F–M**). These results suggest that *EjMYB10* acts as a weak anthocyanin activator.

## 4 Discussion

Commercially cultivated loquats are unable to accumulate anthocyanins in the peel and flesh. However, the accumulation of anthocyanins in the peel and/or flesh enhances the appearance and nutritional quality of loquat fruits. Understanding the mechanism underlying anthocyanin biosynthesis in loquat fruits will provide insights into the development of new cultivars that are rich in anthocyanins. In the present study, we showed that some SP3H loquat fruits accumulate anthocyanins in their red-pigmented peels. We further investigated the mechanism responsible for anthocyanin production in the peel of SP3H loquat fruits.

The composition of anthocyanins varies among species. Cyanidins have been reported to be the most abundant anthocyanins in fruits of rosaceous species, including apple (Feng et al., 2013; Tsao et al., 2003), pear (Pierantoni et al., 2010; Zhang et al., 2020), peach (Cheng et al., 2014; Liu et al., 2019), plum (Fang et al., 2021a, Fang et al., 2025, Fang et al., 2021b; Usenik et al., 2009) and cherry (Liu et al., 2011). In apple, cyanidin-3-galactoside was the most predominant anthocyanin (Espley et al., 2013), while cyanidin-3-glucoside represents the most abundant anthocyanin in peach (Cheng et al., 2014; Jiao et al., 2014; Zhou et al., 2015) and Chinese plum (Fang et al., 2025, Fang et al., 2021b). However, anthocyanins are dominated by cyanidin-3-rutinoside in European plum (Usenik et al., 2009), which was reported to be the dominant anthocyanin accumulated in cherry fruits (Liu et al., 2011). These results suggested the dominant anthocyanin is species-dependent. Our results indicated that the most abundant anthocyanin in the SP3H loquat peel is cyanidin-3-O-galactoside, which was identified as that dominant anthocyanin in *E. henryi* fruits in our previous study (Su et al., 2023). The anthocyanin composition in other red-pigmented fruits of *Eriobotrya* species, such as *E. seguinii*, remain to be investigated.

In this study, we found that SP3H loquat fruits accumulate anthocyanins in the peel. Although the exact conditions required for inducing anthocyanin accumulation in SP3H loquat fruits are unclear, we speculate that this could be a result of environmental stimulus such as high light. We observed that only peels exposed to direct sunlight were able to accumulate anthocyanins. This suggests that light is indispensable for anthocyanin accumulation in loquat peels. However, it is noteworthy that not all fruits exposed to direct sunlight accumulate anthocyanins, and this phenomenon does not occur in SP3H loquat fruits every year. These observations suggest that the conditions required for inducing anthocyanin accumulation in loquat peels are complicated. Environmental and other factors, such as light, temperature, hormone, nutrients and mechanical damage have been demonstrated to induce anthocyanin accumulation via modulating the transcription of genes that participate in anthocyanin biosynthetic and transport in fruits (An et al., 2019; Espley and Jaakola, 2023; Fang et al., 2025; Wang et al., 2023; Zhao et al., 2023). Our previous study showed that most anthocyanin pathway genes were significantly upregulated in the red-pigmented peels of *E. henryi* fruits (Su et al., 2023). However, only nine genes encoding *CHS*, *CHI*, *F3′H*, *ANS* and *UFGT*, as well as a *GST* gene were significantly upregulated in red-pigmented peels of SP3H loquat fruits (**Figure 2**). The significant correlation between anthocyanin content and the expression of structural genes suggests that anthocyanin accumulation in SP3H loquat fruits results from the activation of anthocyanin biosynthetic and transport genes.

The enhanced transcription of anthocyanin pathway genes implies that anthocyanin biosynthesis in the peel of SP3H loquat fruits was transcriptionally regulated. R2R3 MYBs have been proven to be key anthocyanin activators in *Rosaceae* species (Fang et al., 2021b; Gu et al., 2015; Jin et al., 2016; Lin-Wang et al., 2010; Qian et al., 2014; Rahim et al., 2014). RNA-seq and qRT-PCR results indicated that *EjMYB10* was highly expressed in the red-pigmented peel of SP3H loquat fruits (**Figures 3**, **4**). Sequence analysis indicated that *EjMYB10* encodes a R2R3 MYB that is highly homologous to the apple anthocyanin activator MdMYB110a (Umemura et al., 2013) (**Figure 5**). Overexpression of *EjMYB10* and plum *PsbHLH3* resulted in anthocyanin production and red coloration in tobacco leaves (**Figures 6A, B**). Dual-luciferase assays also showed that EjMYB10 enhanced transcriptional activity of anthocyanin biosynthetic genes *EjF3′H*, *EjANS* and *EjUFGT* (**Figures 6F–M**). These results suggested that EjMYB10 is an anthocyanin activator. It is noteworthy that the concentration of anthocyanin pigments induced by *EjMYB10* and *PsbHLH3* was comparable to that induced by the weak activator *PsMYB10.2* (Fang et al., 2021b) but much less than that induced by the strong activator *PsMYB10.1* (Fang et al., 2021a). These results suggest that *EjMYB10* is a weak anthocyanin activator.

## 5 Conclusions

In this study, the mechanism responsible for the red pigmentation in the peel of SP3H loquat was investigated using metabolomic and transcriptomic analyses. Our results showed that anthocyanin accumulation is responsible for the red coloration of loquat peel and the predominant anthocyanin was cyanidin-3-O-galactoside. RNA-seq revealed that anthocyanin accumulation in loquat peel was associated with the high expression of anthocyanin structural genes and the transcription factor *EjMYB10.* Transient overexpression and dual luciferase assays showed that *EjMYB10* can induce weak anthocyanin accumulation in tobacco leaves when co-expressed with *PsbHLH3* and activate the promoters of *EjF3′H*, *EjANS*, and *EjUFGT*. In summary, the results presented here offer novel perspectives on the molecular mechanism underlying anthocyanin biosynthesis in the peel of SP3H loquat. Further studies should investigate the conditions for inducing red coloration and identify genes that regulate the transcription of *EjMYB10*.

## Data Availability

The datasets presented in this study can be found in online repositories. The names of the repository/repositories and accession number(s) can be found in the article/**Supplementary Material**.
