# Supplementary figures and images for: Metabolomic and transcriptomic analyses provide insights into the red pigmentation in loquat (Eriobotrya japonica) peel
Source: Front Plant Sci. 2025 Jun 18;16:1615281. doi: 10.3389/fpls.2025.1615281 (PMC12213514; doi:10.3389/fpls.2025.1615281)

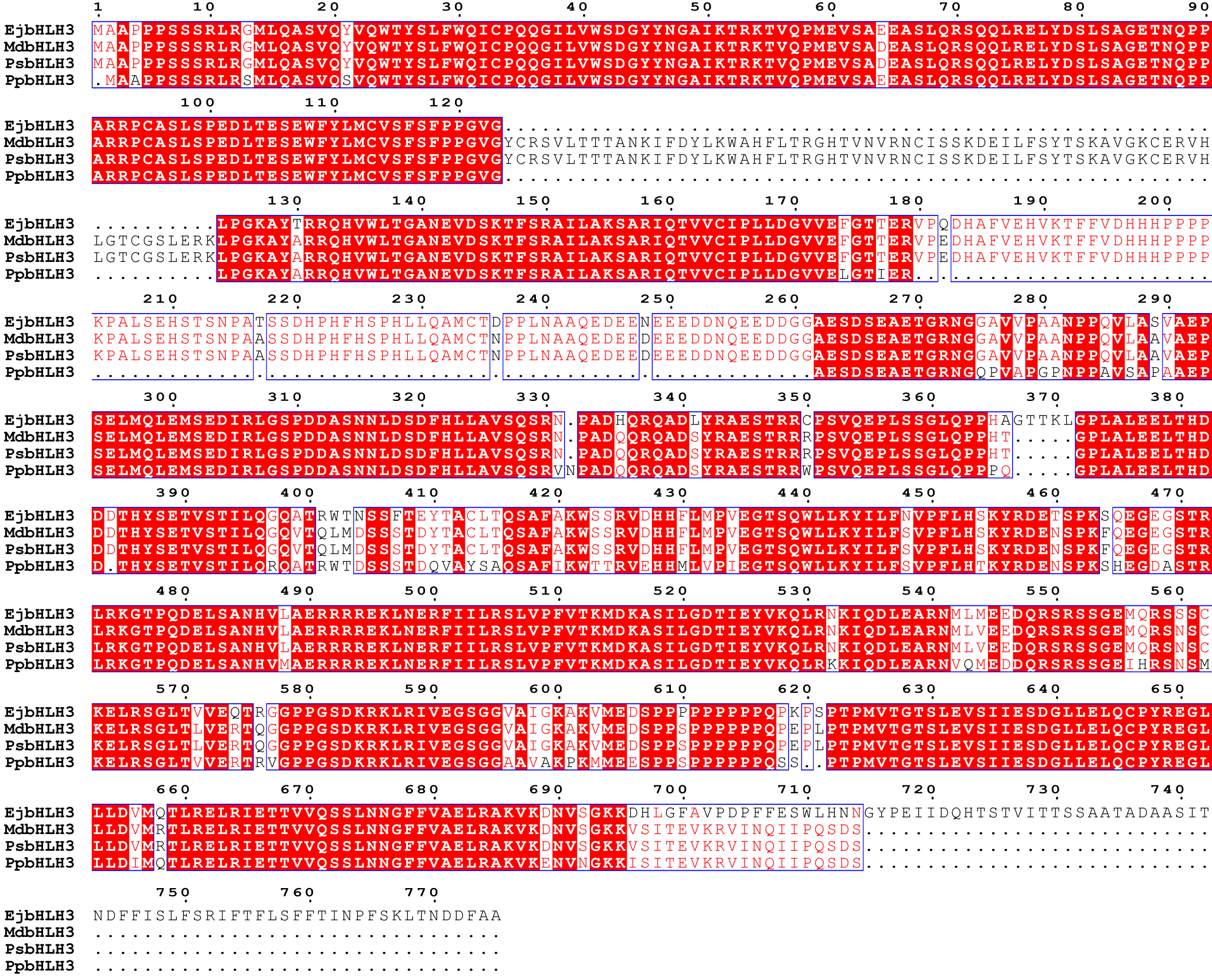

Supplement: Supplementary file 1 [file Image1.tif]

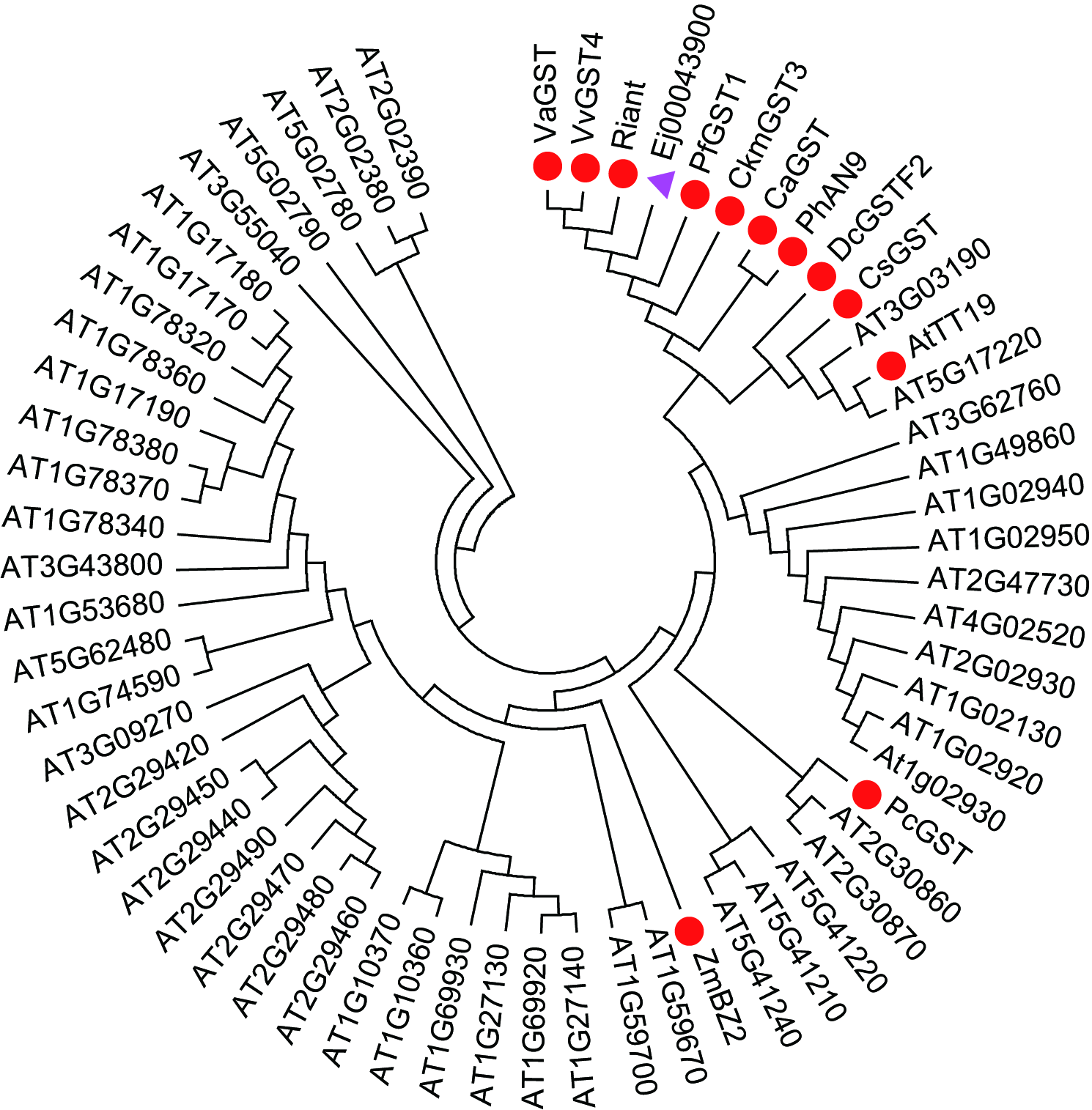

Supplement: Supplementary file 2 [file Image2.tif]
