# Supplementary material for: Metabolomic and transcriptomic analyses provide insights into the red pigmentation in loquat (Eriobotrya japonica) peel
Source: Front Plant Sci. 2025 Jun 18;16:1615281. doi: 10.3389/fpls.2025.1615281 (PMC12213514; doi:10.3389/fpls.2025.1615281)
Supplement: Supplementary file 3 [file Table1.doc]

**Table S1.** Primer sequences for qRT-PCR analysis.

| Genes | Gene ID | Forward primer | Reverse primer |
| --- | --- | --- | --- |
| *CHS* | Ej00014264 | TCGAGTGCGTGTGTGCTT | GCACACCCCACTCCAGTC |
| *CHS* | Ej00014465 | CGGCAGGACATGGTGGTT | CATGTCGACACCGCTGGT |
| *CHI* | newGene15317 | GGGCTCCTCCAACACTTTGT | ACTCAACGGACTCCGACAAC |
| *F3’H* | Ej00025308 | GGCGAAGACCCAAAGGCT | CACCCCCTGCAAGTCCAG |
| *ANS* | newGene13949 | GGGCCTCAAGTTCCCACC | GAGCTCGTCGGAGATGCC |
| *UFGT* | Ej00006885 | TCGTGTCACATTGCGGCT | CCCAACACGTCCTCCACC |
| *UFGT* | Ej00084941 | AAGGAATCGGTTGGCGGG | GCGGCCAAGCAATCATCG |
| *MYB10* | Ej00002033 | ATCAGACCTCAACCCCGAAG | TGTCGACGACGACGTTTGTG |
| *ACTIN* | Ej00095133 | CTTTCCCTCTATGCCAGTG | CAAGGTCAAGCCTCAAGAT |
