# Supplementary material for: Metabolomic and transcriptomic analyses provide insights into the red pigmentation in loquat (Eriobotrya japonica) peel
Source: Front Plant Sci. 2025 Jun 18;16:1615281. doi: 10.3389/fpls.2025.1615281 (PMC12213514; doi:10.3389/fpls.2025.1615281)
Supplement: Supplementary file 5 [file Table3.doc]

**Table S3.** Sequences of the primers used for gene cloning and vector construction.

| Primer name | Sequence | Description |
| --- | --- | --- |
| EjMYB10-F | actagtggatccaaagaattcATGGAGGAATATAACGTTAACTTTAGTGTG | Primers used for inserting *EjMYB10* into vector pSAK277 |
| EjMYB10-R | agaagtactctcgagaagcttCTAACTCAAGACTGGGACATGCAG |
| pF3′H-F | gtcgacggtatcgataagcttCAGCTCGTCTTCGTAGCAAGG | Primers used for inserting pF3′H into vector pGreenII LUC+ |
| pF3′H-R | agtggatcccccgggctgcagGGCGTTTAGATTGTGTGTGGAG |
| pANS-F | gtcgacggtatcgataagcttGTCAACGATCGAAATTGGTCAC | Primers used for inserting pANS into vector pGreenII LUC+ |
| pANS-R | agtggatcccccgggctgcagGAATTCACTGAATCAGAACTCACC |
| pUFGT-F | gtcgacggtatcgataagcttGTTGTGACGAGTACATCACGTGT | Primers used for inserting pUFGT into vector pGreenII LUC+ |
| pUFGT-R | agtggatcccccgggctgcagTACAACTTACAATGCTAATTAGGAAAG |
