# Supplementary material for: Metabolomic and transcriptomic analyses provide insights into the red pigmentation in loquat (Eriobotrya japonica) peel
Source: Front Plant Sci. 2025 Jun 18;16:1615281. doi: 10.3389/fpls.2025.1615281 (PMC12213514; doi:10.3389/fpls.2025.1615281)
Supplement: Supplementary file 6 [file Table4.docx]

**Table S4** The accession number of MYBs used for phylogenetic analysis

| Name | accession number | species |
| --- | --- | --- |
| AtMYB75 | AT1G56650 | *Arabidopsis thaliana* |
| AtMYB90 | AT1G66390 | *Arabidopsis thaliana* |
| AtMYB113 | AT1G66370 | *Arabidopsis thaliana* |
| AtMYB114 | AT1G66380 | *Arabidopsis thaliana* |
| FaMYB1 | AAK84064.1 | *Fragaria×ananassa* |
| FaMYB10 | ABX79947.1 | *Fragaria×ananassa* |
| McMYB | MG571450 | *Myrciaria cauliflora* |
| MdMYB1 | XP_028963316.1 | *Malus×domestica* |
| MdMYB10 | EU518249 | *Malus×domestica* |
| MdMYB110a | EB710109 | *Malus×domestica* |
| PhMYB27 | KF985023 | *Petunia hybrida* |
| PcMYB10.1 | KP772281 | *Prunus cerasifera* |
| PcMYB10.2 | KP772282 | *Prunus cerasifera* |
| PpMYB10.1 | XM_007216468 | *Prunus persica* |
| PpMYB10.2 | XM_007216161 | *Prunus persica* |
| PsMYB10.1 | MK105923 | *Prunus salicina* |
| PsMYB10.2 | MK340932 | *Prunus salicina* |
| PyMYB10 | GU253310 | *Pyrus pyrifolia* |
| PyMYB114 | MF489219 | *Pyrus pyrifolia* |
| VbMYBA | MW543447 | *Vaccinium bracteatum* |
