# Supplementary material for: Metabolomic and transcriptomic analyses provide insights into the red pigmentation in loquat (Eriobotrya japonica) peel
Source: Front Plant Sci. 2025 Jun 18;16:1615281. doi: 10.3389/fpls.2025.1615281 (PMC12213514; doi:10.3389/fpls.2025.1615281)
Supplement: Supplementary file 7 [file Table5.docx]

**Table S5 Summary of RNA-Seq data**

| Sample Name | Clean reads | Clean bases | GC Content | %≥Q30 | Map Rate |
| --- | --- | --- | --- | --- | --- |
| Yellow_1 | 21,158,472 | 6,333,361,962 | 46.77% | 90.52% | 95.90% |
| Yellow_2 | 20,141,973 | 6,023,796,006 | 46.75% | 93.23% | 94.97% |
| Yellow_3 | 21,709,048 | 6,500,236,832 | 46.46% | 91.36% | 94.64% |
| Red_1 | 23,032,366 | 6,894,643,758 | 47.26% | 92.27% | 94.37% |
| Red_2 | 21,490,308 | 6,429,793,382 | 46.54% | 90.51% | 95.17% |
| Red_3 | 19,988,682 | 5,984,902,504 | 46.78% | 93.49% | 95.04% |
