# Supplementary material for: Metabolomic and transcriptomic analyses provide insights into the red pigmentation in loquat (Eriobotrya japonica) peel
Source: Front Plant Sci. 2025 Jun 18;16:1615281. doi: 10.3389/fpls.2025.1615281 (PMC12213514; doi:10.3389/fpls.2025.1615281)
Supplement: Supplementary file 8 [file Table6.docx]

**Table S6 Summary of functional annotations for putative new genes**

| Annotated databases | New Gene Number |
| --- | --- |
| COG |  |
| GO | 276 |
| KEGG | 1,156 |
| KOG | 912 |
| Pfam | 579 |
| Swiss-Prot | 954 |
| TrEMBL | 845 |
| eggNOG | 1,601 |
| nr | 1,157 |
| All | 1,624 |
